# Yellow Fever Vaccination in a Mouse Model Is Associated With Uninterrupted Pregnancies and Viable Neonates Except When Administered at Implantation Period

**DOI:** 10.3389/fmicb.2020.00245

**Published:** 2020-02-20

**Authors:** Fernanda C. da Silva, Fernanda M. Magaldi, Helena K. Sato, Estela Bevilacqua

**Affiliations:** ^1^Department of Cell and Developmental Biology, Institute of Biomedical Sciences, University of São Paulo, São Paulo, Brazil; ^2^Secretaria do Estado de São Paulo, Epidemiological Surveillance Center, Department of Health, São Paulo, Brazil

**Keywords:** yellow fever (YF) vaccine, pregnancy, congenital transmission, placenta, fetal losses

## Abstract

The potential risk of yellow fever (YF) infection in unvaccinated pregnant women has aroused serious concerns. In this study, we evaluated the effect of the YF vaccine during gestation using a mouse model, analyzing placental structure, immunolocalization of the virus antigen, and viral activity at the maternal-fetal barrier and in the maternal liver and fetus. The YF vaccine (17DD) was administered subcutaneously at a dose of 2.0 log_10_ PFU to CD-1 mice on gestational days (gd) 0.5, 5.5, and 11.5 (*n* = 5–10/group). The control group received sterile saline (*n* = 5–10/group). Maternal liver, implantation sites with fetus, and placentas were collected on gd18.5. The numbers of implantation sites, reabsorbed embryos, and stillborn fetuses were counted, and placentas and live fetuses were weighed. Tissues (placenta, fetuses, and liver) of vaccinated pregnant mice on gd5.5 (*n* = 15) were paraffin-embedded in 10% buffered-formalin and collected in TRIzol for immunolocalization of YF vaccine virus and PCR, respectively. PCR products were also subjected to automated sequence analysis. Fetal growth restriction (*p* < 0.0001) and a significant decrease in fetal viability (*p* < 0.0001) occurred only when the vaccine was administered on gd5.5. In stillbirths, the viral antigen was consistently immunolocalized at the maternal-fetal barrier and in fetal organs, suggesting a transplacental transfer. In stillbirths, RNA of the vaccine virus was also detected by reverse transcriptase-PCR indicating viral activity in the maternal liver and fetal tissues. In conclusion, the findings of this study in the mouse suggest that vaccination did not cause adverse outcomes with respect to fetal development except when administered during the early gestational stage, indicating the **i**mplantation period as a susceptible period in which the YF vaccine virus might interfere with pregnancy.

## Introduction

Yellow fever (YF) is an acute viral infection associated with hepatitis, jaundice, hemorrhage, and renal failure, and it may progress to death (Monath, 2008; Staples and Monath, 2011). Acute febrile syndrome and hemorrhagic phenomena are the most prominent manifestations of this disease. Vaccination and strategic programs for controlling the main vectors—hematophagous mosquitoes of the genera *Aedes, Haemogogos*, and *Sabethes*—have been successfully implemented in many countries, even though the number of infected persons has increased worldwide over the last two decades (Monath, 2013; Monath and Vasconcelos, 2015). Large outbreaks have been reported in Africa as well as South and Central America, mainly in forested areas infested by the transmission vectors (Staples and Monath, 2011).

In Brazil, no urban outbreak of YF has occurred since the 1940s, when the urban cycle of transmission was eradicated. However, in 1997, YF viral infections were detected in North and Central Brazil, and a new outbreak was registered in 2008 in South and Southeast regions of the country (Monath and Vasconcelos, 2015; Waggoner et al., 2018). In 2017, YF virus (YFV) was detected in non-human primates, and human cases were reported in places where vaccination coverage was low (Leal et al., 2016; Fernandes et al., 2017), a situation that may have been aggravated by public hesitancy toward vaccines.

YF virus are single-stranded RNA viruses of the Flaviviridae family. The direct cytopathic effect of the virus and a potent host immune response are associated with the secretion of TGF-b, TNF-a, and IFN-g, which are believed to play roles in the manifestations and severity of the disease (Quaresma et al., 2006a, b, 2013; Woodson et al., 2011; Engelmann et al., 2014). Transcriptomic analyses of viscerotropic YF in a rhesus macaque model also showed that YF infection correlates with changes in cytokine gene expression before the emergence of clinical symptoms, suggesting these immune factors (cytokines) may influence the disease outcome (Engelmann et al., 2014). The results of *in vitro* studies have suggested that effects of infection on endothelial cells may also contribute to YF pathogenesis (Khaiboullina et al., 2005; Quaresma et al., 2006a). Inoculation of flaviviruses in monkeys indicates that the initial site of replication is the inoculation site in the skin followed by the lymph nodes, where additional replication occurs (Marchette et al., 1973; Monath and Barrett, 2003; Dean et al., 2005). The virus reaches the bloodstream via the lymphatic system and spreads to prevalent organs, such as the liver (Monath and Vasconcelos, 2015). In the liver, the virus infects the Kupffer cells and the hepatocytes, causing severe necrosis (Monath and Barrett, 2003).

The YF vaccine virus is a live attenuated virus, which is prepared and obtained by culturing the 17D strain virus in living chick embryos, and it can induce neutralizing antibodies and T lymphocyte responses. The vaccine differs from YFV by the loss of viscerotropism, despite its replicative activity in cell culture, and by 20 amino acid changes in the envelope protein (Lee and Lobigs, 2008; Monath et al., 2013). In monkeys, the YF 17D vaccine causes a transient low viremia (Monath and Barrett, 2003) accompanied by innate immune responses with detectable levels of cytokines and toll-like receptor-mediated signaling (Monath and Vasconcelos, 2015). Neutralizing antibodies are the principal mediators of protective immunity against flaviviruses (Monath et al., 2013).

After a single dose of the YF 17D vaccine, 80–90% of human subjects become seropositive by day 10 (Monath and Barrett, 2003). A small number of adverse events have been associated with vaccination in humans and monkeys, ranging from severe encephalitis and hepatic failure to neurological symptoms of benign prognosis (Monath and Barrett, 2003; Martins et al., 2015).

In pregnant women, Nishioka et al. (1998) found a relative risk of 2.29 for spontaneous abortions after vaccination. Another study performed on women who inadvertently received YF vaccine during pregnancy reported abortion, stillbirth, and malformation rates similar to those found in the general population (Nasidi et al., 1993; Robert et al., 1999; Suzano et al., 2006; D’Acremont et al., 2008). Nonetheless, due to the theoretical risk of maternal-fetal transmission associated with fetal hepatic and neuronal susceptibility to the YF 17D virus, there is a general recommendation to avoid vaccine administration during pregnancy except when epidemiologically justified (Hagmann et al., 2017). However, the absence of vaccination during pregnancy is a risk to the mother and fetus, thereby increasing the risk of infection to local mosquitoes. Therefore, it is imperative to disseminate knowledge and awareness on gestational vaccination.

A small number of viruses are transmitted from mother to fetus, showing the effectiveness of the hemochorial barrier against these infections (Marinho et al., 2017). However, the mechanisms by which the viruses overcome the placental barrier is still uncertain. Recent studies suggest that maternal immunity, time of gestation, coinfections, and many other factors may be associated with this effectiveness (Marinho et al., 2017).

In this study, we used a mouse model to analyze the effect of YF vaccination during gestation and to identify potential susceptible phases that might compromise embryo/fetal health. This study addressed the birth/mortality rates, as well as the morphology and localization of YF 17D virus in fetuses, placentas, and maternal tissues after vaccination through immunohistochemical reactions and polymerase chain reaction (PCR).

## Materials and Methods

### Animals

Female adult mice (CD-1 mice, 3 months old) were caged overnight with males (1:1) and successful mating was verified the following morning. The presence of a vaginal plug indicated day 0.5 of gestation (gd). All animal care and experimental procedures were carried out according to the Brazilian Society of Science in Laboratory Animals (COBEA) and was approved by the Ethics Committee for Animal Research (CEEA) of Biomedical Sciences Institute of the University of São Paulo, Brazil.

### Vaccination

The vaccination protocol was carried out at the Department of Cellular and Developmental Biology in the Biomedical Sciences Institute of the University of São Paulo, under the supervision of the Department of Immunization Center for Epidemiological Surveillance of the State of São Paulo. YF vaccine (17DD, parts 00PVFA028Z; 066VFA061Z; and 082VFB006Z) was obtained from the Oswaldo Cruz Foundation (Bio-Manguinhos, Rio de Janeiro, Brazil). The vaccine was reconstituted with 5 mL of saline and administered subcutaneously at a dose of 2.0 log_10_ PFU in a final volume of 0.1 mL. In the control group, the vaccine was replaced by sterile saline (PBS, Gibco BRL, Grand Island, NY, United States).

### Experimental Design

The experiments were divided into two phases. In the first phase, vaccination was carried out at different stages of pregnancy (0.5, 5.5, and 11.5 gestation days [gd]) for gestational parameter evaluation (*n* = 5–10 pregnant females/experimental or control group). Day 0.5 of gestation is a period when the zygote is in the lumen of the uterine tube; day 5.5 of gestation represent the onset of implantation, in which the trophoblast giant cells surrounding the blastocyst come into direct contact with the maternal blood; and day 11.5 of pregnancy is the usual time for placenta maturation (Adamson et al., 2002; Cross, 2005; Watson and Cross, 2005; Hu and Cross, 2009; Croy et al., 2014).

In the second phase, immunolocalization of the viral antigen and signals of viral activity were analyzed at periods in which the vaccination had caused relevant changes (gd5.5, *n* = 15 pregnant females/experimental groups and *n* = 10/control groups).

### Sample Collection for Gestational Performance

Vaccinated and control animals were anesthetized with hydrochloride xylazine (Rompun 2%^®^, Bayer, São Paulo, Brazil) and ketamine (1:1, v/v, Ketalar, Bayer, São Paulo, Brazil) on gd18.5. Uterine horns were dissected under a stereoscopic microscope, and implantation sites with fetuses and their placentas were exposed. The fetuses were euthanized in a CO_2_ chamber. Placentas, resorptions, and living and dead fetuses were counted and weighed. The total number of implantation sites and early reabsorbed embryos was evaluated by incubating the uterine horns in 10% ammonium sulfide for 10 min after the removal of the fetuses, placentas, and late resorptions (Salewsky, 1964).

Birth index (BI) and mortality rate (MR) were respectively calculated as follows:

$$\mathrm{BI}=\frac{\begin{array}{c} \mathrm{Total}\,\mathrm{number}\,\mathrm{of}\,\mathrm{implantation}\,\mathrm{sites}-\\ \mathrm{number}\,\mathrm{of}\,\mathrm{stillbirths}\,\mathrm{and}\,\mathrm{resorptions} \end{array}}{\mathrm{Total}\,\mathrm{number}\,\mathrm{of}\,\mathrm{implantation}\,\mathrm{sites}}$$

$$\mathrm{MR}=\frac{\begin{array}{c} \mathrm{Total}\,\mathrm{number}\,\mathrm{of}\,\mathrm{implantation}\,\mathrm{sites}-\\ \mathrm{number}\,\mathrm{of}\,\mathrm{live}\,\mathrm{fetuses} \end{array}}{\mathrm{Total}\,\mathrm{number}\,\mathrm{of}\,\mathrm{implantation}\,\mathrm{sites}}\times 100$$

Results were expressed as the mean value ± SD, and Student’s *t*-test was used to determine significant differences in comparison with age-control groups. A probability level of less than 5% was considered significant. Statistical analysis was performed using the program Statistical Package for Social Science for Windows.

### Collection and Processing of Samples for Morphological Analysis

Under deep anesthesia, fragments of the maternal liver and the uterine horns were obtained. Placentas, fetuses (live fetuses were euthanized by CO_2_ inhalation) and material resulting from resorptions were then dissected. Tissues were fixed in 10% buffered formalin and routinely processed for embedding in Histosec^®^ (Merck KGaA, Darmstadt, Germany). Sections were either stained with hematoxylin and eosin or processed for immunoreactions and light microscopy analysis.

Sections of the maternal liver, placenta, and fetus of control females (*n* = 5) and vaccinated mothers with living (*n* = 5) and dead (stillbirth, *n* = 5) fetuses obtained from different mothers were assessed with immunohistochemical (IH) assays. At least three sections from each placenta (three placentas per animal) and of maternal liver and fetus were obtained from each experimental animal for analysis. Deparaffinized and hydrated sections were incubated in the citric acid solution (10 mM, pH 6.0) for 3 min at 60°C and thereafter blocked for 15 min in 3% hydrogen peroxide in distilled water. Sections were incubated for 1 h in M.O.M. mouse IgG blocking reagent (Vector Lab, Burlingame, CA, United States). Next, the samples were immunostained using the polyclonal mouse anti-YF virus antibody (Division of Medical Biology, Department of Virology, Adolfo Lutz Institute, São Paulo, Brazil) diluted at 1:2,000 in TBS containing 1% bovine serum albumin, for 30 min at 37°C, followed by 18 h at 4°C. Labeled polymer-HRP anti-mouse (EnVision + System HRP [DAB], Dako Cytomation) was used as a secondary antibody for 1 h at room temperature. Color development was obtained by incubating with DAB substrate-chromogen solution (0.05% 3,3′- diaminobenzidine in hydrogen peroxide, Sigma-Aldrich, St. Louis, MO, United Ststes) for 5 min. Sections were counterstained with Mayer’s hematoxylin and examined using an Axioskop 2 light microscope (Carl Zeiss, Oberkochen, Germany). The images were captured with Axio Vision 4.7 software (Carl Zeiss, Oberkochen, Germany). Negative control was performed by omission of the primary antibody and/or by replacing this antibody with non-immune serum. The sensitivity of the reaction was tested by using immunoreactive liver samples of a patient diagnosed with YF who had a known expression of the viral antigen (positive control).

### Detection of Viral RNA by RT-PCR

YF virus envelope protein gene fragments were detected through the PCR assay in maternal liver, placenta, fetal brain, and liver of live fetuses and stillbirths of vaccinated mothers on gd5.5 and in the placenta and liver of mothers vaccinated on gd0.5 and 11.5. Positive control reactions were performed using samples of the YF vaccine. Total RNA was isolated with TRIzol (Invitrogen^TM^, Carlsbad, CA, United States) and suspended again in sterile distilled water according to Chomizynski and Sacchi (1987). All reagents were purchased from Sigma Aldrich (St. Louis, MO, United States), unless otherwise stated. RNA concentration and purity were determined by spectrophotometric measurement of absorbance at 260 nm, and the purity was determined at A260/A280 ratio. The RNA integrity was checked by using 1% agarose gel electrophoresis with 0.4 mol/L Tris-acetate and 0.001 mol/L EDTA buffer. Viral RNA was converted to cDNA using 5.0 μg of RNA, 5.0 μL of specific antisense primer (5′-GCT TTT CCA TAC CCA ATG AA-3′ (MG922934.1), 2.0 μL dNTPs Mix, 0.75 μL M-MLV reverse transcriptase (Invitrogen^TM^), 6.0 μL reaction buffer 5 × and 3.0 μL DTT 0.1 M. The mixture was incubated at 37°C for 90 min and at 95°C for 5 min to inactivate the reverse transcriptase. The viral cDNA (6.0 μL) was amplified by PCR using a 2.5 μL 10 × PCR buffer (Biotools B&M Labs S.A., Madrid, Spain), 2.0 μL MgCl_2_ (50 mM), 1.0 μL of dNTPs Mix, 0.5 μL DMSO 4%, 0.75 μL DNA polymerase (1 U/μL, Biotools B&M Labs S.A., Madrid, Spain), and 5.0 μL (10 pmol/μL) each of the forward and reverse primers (**1**: 5′-TAC CCT GGA GCA AGA CAA GT-3′; **2**: 5′-GCT TTT CCA TAC CCA ATG AA-3′). The PCR was performed in a Bio-Rad Gene Cycler^TM^ (Bio-Rad Laboratories, Portland, ME, United States). Cycling conditions included denaturation at 94°C for 5 min, 35 PCR cycles of 94°C for 1 min, 58°C for 2 min, 72°C for 3 min and the last step for a final extension at 72°C for 10 min in a thermocycler. PCR was performed using reverse transcripted products from the vaccine’s RNA as a template. PCR products were analyzed on 1% agarose gel electrophoresis using a molecular weight marker DNA (100 base pairs, DNA Ladder, Ludwig Biotec, Nova Alvorada, Brazil) as reference. The gel was exposed to a Molecular Imaging screen (G: Box Chemil-R, Syngene, Frederick, MD, United States) for computerized gel documentation (Scion image program, Scion Corp., Frederick, MD, United States). The identity of the 482 bp-amplified products was confirmed by sequence analysis (automated sequence analysis, MegaBACE 1000, GE Healthcare, Buckinghamshire, United Kingdom) at the Center for Human Genome Studies at the University of São Paulo (Brazil). Fluorograms were analyzed using the Cimarron 3.12 base-caller software. A sequence database search was performed using the BLAST network service of the National Center for Biotechnology Information^1^.

## Results

Vaccination on gd0.5 and 11.5 did not affect the average number of implantation sites and fetal resorption per pregnant female in comparison to controls (Table 1). In the group that received vaccination on gd5.5, the mean number of degenerated fetuses and stillbirths and the number of embryo/fetal losses significantly increased (from 0.13 in control group to 1.37 in vaccinated group, *p* = 0.01; and from 0.38 in controls to 3.5 in vaccinated animals, *p* = 0.002, respectively), resulting in an increased mortality rate (from 5.1% in control group to 33.1% in vaccinated group, *p* = 0.001) in comparison to sham controls, as shown in Table 1.

**TABLE 1:** Effect of anti-yellow fever vaccination administered on gestation days 0.5, 5.5, and 11.5 on the gestational parameters.

	***n***	**Implantation sites**	**Number of fetuses**	**Degenerated fetuses/stillbirths**	**Embryo/fetal losses**	**Mortality rate (%)**
Control gd 0.5	56	11.2 ± 1.64	10.6 ± 1.95	0.4 ± 0.55	1.0 ± 1.2	9.2 ± 10.3
Vaccinated gd 0.5	53	10.6 ± 3.71	10.0 ± 4.06	0.4 ± 0.89	1.0 ± 1.0	11.6 ± 11.1
Control gd 5.5	96	12.0 ± 2.33	11.7 ± 2.25	0.13 ± 0.35	0.38 ± 0.52	5.1 ± 7.3
Vaccinated gd 5.5	97	12.1 ± 1.89	9.5 ± 1.77	1.37^a^ ± 1.19	3.5^b^ ± 1.69	33.1^c^ ± 17.1
Control gd 11.5	83	10.3 ± 2.38	9.8 ± 2.23	0.25 ± 0.46	0.75 ± 1.16	6.7 ± 9.9
Vaccinated gd11.5	54	10.8 ± 2.17	10.0 ± 2.65	0.4 ± 0.55	1.2 ± 1.10	7.6 ± 9.0

*n, Total number of implantation sites. Values correspond to mean number per pregnant female ± SD; ^*a*–*c*^highlights statistical differences in comparison to the respective age-control. ^*a*^*p* = 0.01; ^*b*^*p* = 0.002; ^*c*^*p* = 0.001 (Student’s *t*-test).*

Fetal weight gain was significantly lower only when the vaccine was administered in pregnant animals on gd5.5 (*p* = 0.004; Table 2). Placental weight was not altered significantly in any vaccinated group compared to the control group. These data indicate the susceptible period to YF vaccination during pregnancy, justifying the subsequent experimental procedures on only day 5.5 of gestation.

**TABLE 2:** Effect of anti-yellow fever vaccination administered on gestation days 0.5, 5.5, or 11.5 on placental and fetal weights.

	***n***	**Fetal weight (g)**	**Placental weight (g)**
Control gd 0.5	53	0.89 ± 0.17	0.15 ± 0.03
Vaccinated gd 0.5	50	0.87 ± 0.09	0.14 ± 0.02
Control gd 5.5	180	0.95 ± 0.10	0.14 ± 0.04
Vaccinated gd 5.5	158	0.82^a^ ± 0.12	0.13 ± 0.01
Control gd 11.5	83	0.91 ± 0.11	0.13 ± 0.02
Vaccinated gd 11.5	57	0.88 ± 0.06	0.13 ± 0.02

*Values correspond to mean ± SD; values indicated by letters highlight statistical differences in comparison to the respective age-control. Student’s *t*-test (^*a*^*p* = 0.004).*

**FIGURE 1:**
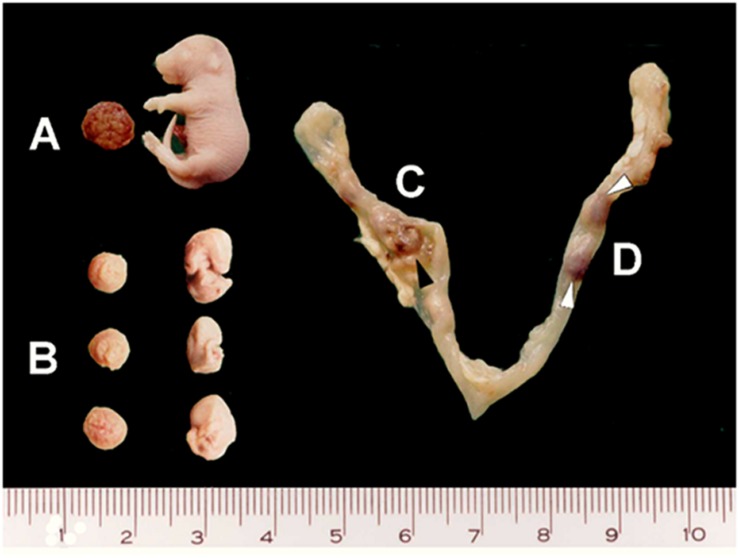
Macroscopic fetal features on gd18.5. Samples from vaccinated pregnant mice present different fetal development, as shown in **(A)** apparently normal (living and stillborn) fetuses, **(B)** early-dead fetuses and **(C,D)** resorptions (black and white arrowheads).

Fetuses were macroscopically divided into live fetuses, stillbirths (absence of heartbeat, but no visible degeneration and therefore, considered as late-dead fetuses), early-dead fetuses (with apparent developmental delay and degenerative signals), and post-implantation resorptions (implantation site with no recognizable fetal structures) (Figure 1). The subsequent analyses were performed on live fetuses and stillbirths.

**FIGURE 2:**
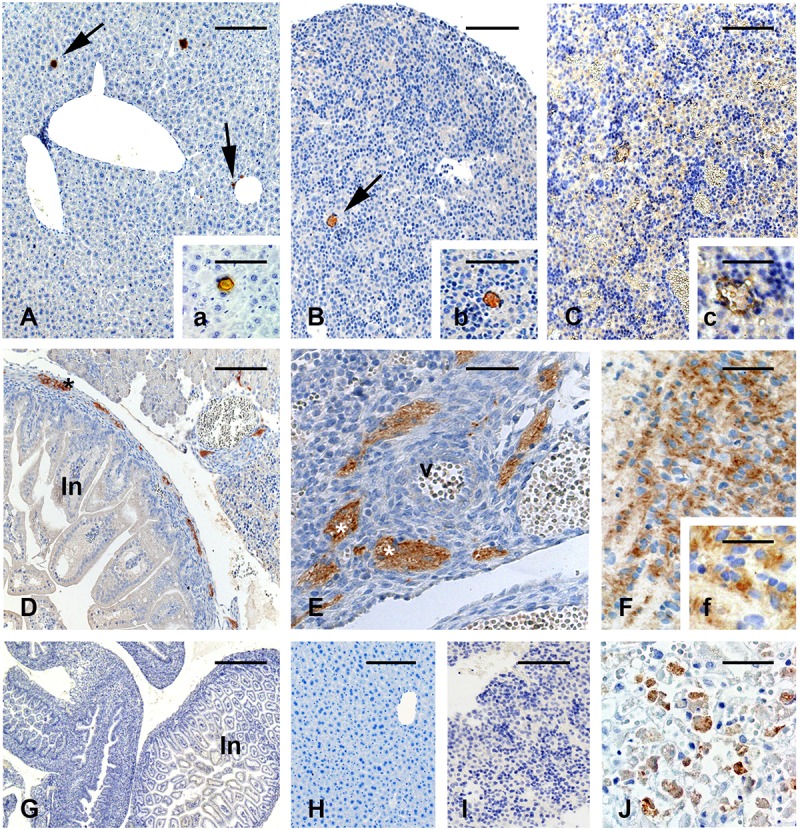
Immunolocalization of the yellow fever (YF) antigen after vaccination on gd 5.5. **(A–C)** In maternal and fetal liver, immunoreactive areas are rare **(A,a)**. Liver of one vaccinated pregnant female **(B,b)**. Liver of a live fetus **(C,c)**. Liver of a stillborn. Reactivity is characterized by a brown colored (arrows) **(D–F)**. Immunolocalization of YF antigen in stillbirths shows reactivity for the virus vaccine in nervous cells in the peripheral area of the developing digestive tract **(D)** and in nerves **(E)** surrounding one arterial vessel (v). Strong reactivity can also be seen in the brain **(F,f)**. Figures **(G–I)** are negative controls of the reaction, in which the primary antibody was replaced by non-immune serum. Figure **(J)** shows the positive control of the IH reaction (liver of a patient with YF). Bars in **(A)** = 150 μm, in **(a,b**,**E)** = 100 μm, in **(B,C,H,I)** = 200 μm, in **(c,F)** = 60 μm, in **(D)** = 350 μm, in **(f)** = 50 μm, in **(G)** = 600 μm, in **(J)** = 75 μm.

In the maternal liver as well as in the liver of live fetuses of the vaccinated females on gd5.5, the viral antigen was rarely immunolocalized (Figures 2A,B,a,b). For IH reactions, no immunolabeling was detected in the non-vaccinated group (not shown) in the negative control, which was performed by omitting the primary antibody (Figures 2G–I**missing**). Biopsies of human liver diagnosed with YF were used as positive control for the IH reactions (Figure 2J).

Unlike live fetuses, reactivity to YFV in the stillbirths was intense and distributed in the cells of several organs (Figures 2C–F). Reactivity was seen in the liver (Figures 2C,c), nervous tissue in the developing intestine (Figure 2D), cells surrounding arterial vessels (Figure 2E), and in the fetal brain (Figures 2F,f).

**FIGURE 3:**
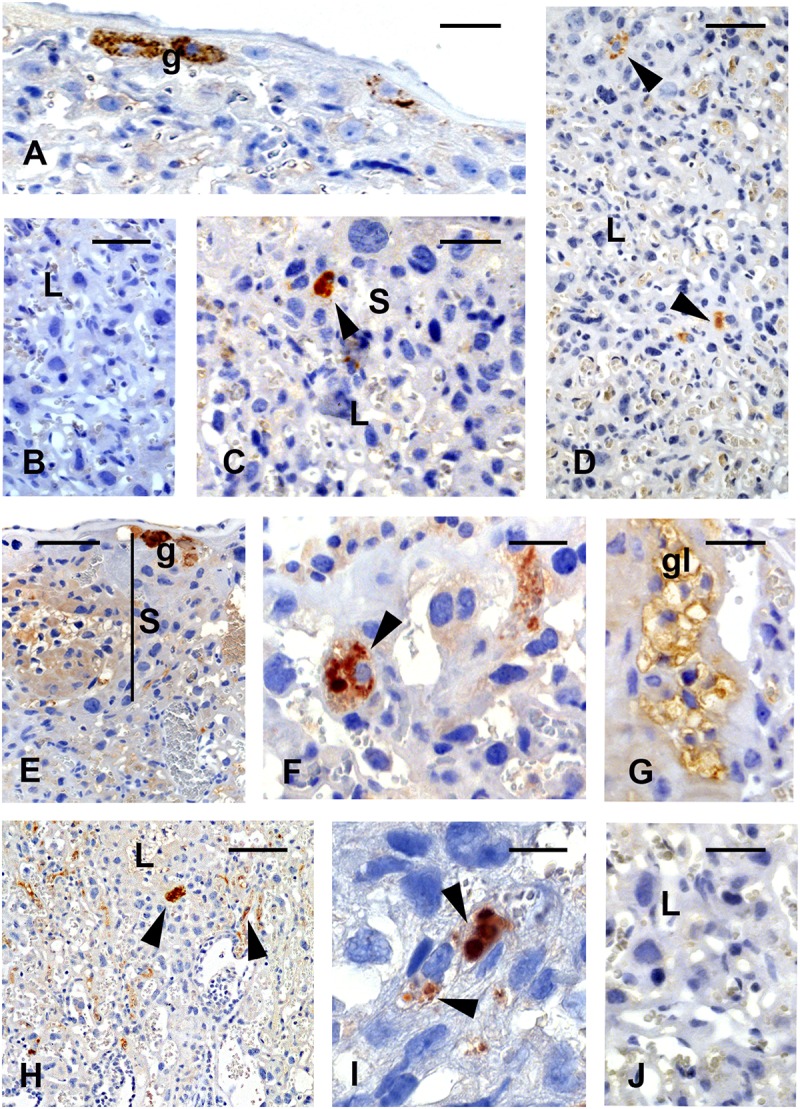
Immunolocalization of the YF antigen after vaccination on gd5.5. Placentas of live fetuses **(A–D)** and stillbirths **(E–J)**. Reactions are characterized by brownish color. The viral antigen is seen at the junctional zone in trophoblast giant cells **(A, g)** and **(C**, arrowhead) in cells of the spongiotrophoblast (S) area. Few cells **(D**, arrowheads) reacted with the antibody against YF virus (YFV) in the labyrinthine region. In stillbirths, the immunoreaction in spongiotrophoblast (S) is intense, in giant cells **(E, g)**, spongiotrophoblast cells **(F**, arrowhead) and in glycogen cells **(G, gl) (H,I)**. Reactivity is also seen in the labyrinth (L, arrowheads). Figures **(B,J)** are negative controls of the reaction. Bars in **(A,D,E)** = 120 μm, in **(B)** = 100 μm, in **(C,G,J)** = 80 μm, in **(F)** = 60 μm, in **(H)** = 240 μm, in **(I)** = 25 μm.

Histological analysis of placentas from control and viable fetuses of vaccinated animals showed typical morphological features. In live fetuses, immunoreactions revealed the presence of the viral antigen in the trophoblast cells of the junctional zone (trophoblast giant cells, Figure 3A) and spongiotrophoblast cells (Figure 3C), and only occasionally in the cells of the labyrinthine layers (Figure 3D).

In general, placentas from stillbirths showed common characteristics in relation to age-control placentas from vaccinated and control living fetuses. Occasional morphological changes found in this group included the scattering of glycogen cell clusters toward the labyrinthine zone. Viral antigen was seen in the trophoblast giant cells (Figure 3E), the spongiotrophoblast cells (Figure 3F), and the glycogen cells (Figure 3G). Reactivity was stronger in the labyrinthine area (Figures 3H,I) in different cell types. The negative IH control did not show any reaction in these tissues (Figures 3B,J).

**FIGURE 4:**
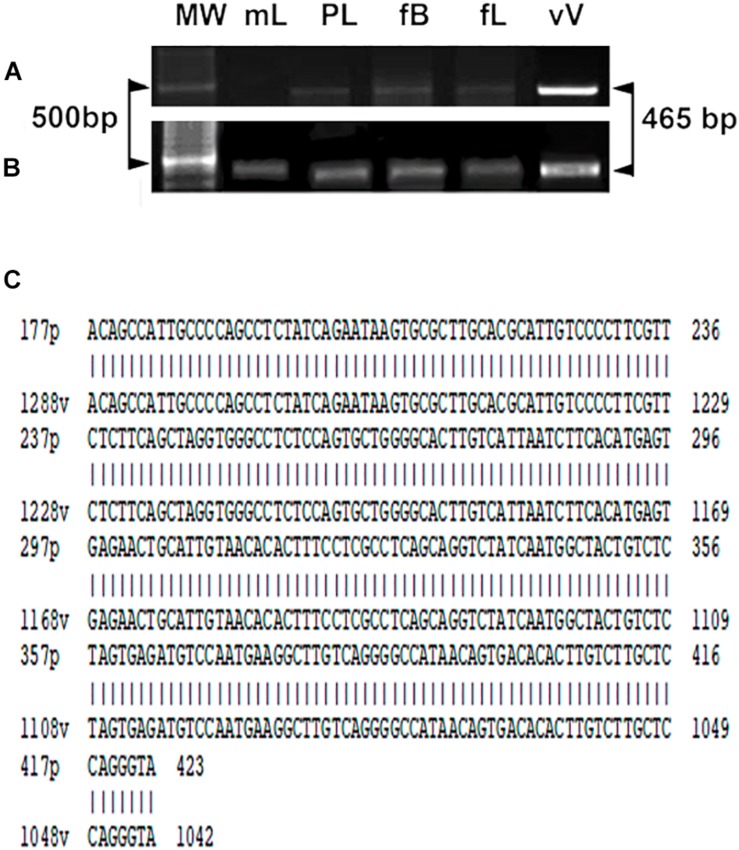
Gene expression of the YF viral capsid protein (465 bp) in maternal liver and tissues of viable fetuses **(A)** and stillbirths **(B)**. Maternal liver (mL), placenta (Pl), fetal brain (fB), liver (fL), (Vv) vaccine YF virus (positive control). The first band of each gel corresponds to the DNA weight in which 500 base pairs are highlighted (arrowheads). **(C)** Sequencing of the experimental PCR product obtained from brain of stillbirths. Note the alignment and 100% identity with the yellow fever virus strain 17DD-Brazil.

The YF vaccine was also detected by RT-PCR using the YFV consensus primer pair. RT-PCR analysis of YFV produced amplicons, as shown in Figures 4A,B, in maternal liver, placenta, fetal brain, and liver in stillbirths. In live fetuses, only traces of the amplicons were found in part of the samples (in tissues of 2 fetuses from 7 analyzed). DNA amplification was not observed in the negative control nor in samples from vaccination on gd0.5 and 11.5 (not shown). YFV identity was confirmed by sequencing PCR products obtained from the brain samples of stillbirths (*n* = 3). The sequence was aligned to the YFV strain 17DD-Brazil, and 100% identity was observed (Figure 4C).

## Discussion

We examined the effect of vaccination against YFV at three different gestational periods in mice and found clear evidence of changes in pregnancy outcomes and fetal infection only when the vaccine was administered during the embryo implantation period (gd 5.5). Vaccination on this particular day of gestation led to increased embryo/fetal losses and growth restriction in live fetuses. The vaccine virus was only occasionally observed in the liver of the live fetuses but consistently found in stillbirths’ tissues. Immunolocalization of the vaccine virus was detected in the placenta of both live fetuses and stillbirths, but was more intense in samples of the stillborn fetuses. The presence of the virus in the placenta and fetal organs in the stillbirths indicated that a vertical passage of the vaccine virus may have occurred. In living fetuses, by contrast, the presence of the virus only in the placenta suggests the maternal-fetal interface may assume a protective role. The differences in the fate of individuals belonging to the same litter, however, are not clearly understood.

Genetic background of each individual may be a factor to be considered, as the strain used here is an outbred mouse. In humans, it has been suggested that modulation of susceptibility to the virus may occur as a result of genetic variation, particularly in loci encoding innate immune mediators (Blake and Garcia-Blanco, 2014). In addition, mice generally have an innate resistance to flavivirus-induced mortality/morbidity due to the autosomal dominant *Flvr* allele. When infected, mice exhibit low levels of viral titers in their tissues, which is associated with a low mortality rate (Nathanson and Brinton, 2007). While that might be one contributing factor, there is also evidence suggesting that the transplacental transfer of a flavivirus is a stochastic process (Yuan and Allen, 2011; Björnberg et al., 2014). Stochastic models have been developed to explain the dynamics of viral infection/transmission, which includes random transitions between infection, latent infection, or non-infection in cells, tissues, and organisms (Tuckwell and Le Corfec, 1998; Yuan and Allen, 2011; Björnberg et al., 2014).

A key question in this study is why changes occurred only following the administration of the vaccine on gd5.5. A plausible explanation may be the immaturity of the cells that comprise the maternal-fetal barrier at this stage of gestation. Day 5.5 of gestation marks the beginning of the implantation process. At this time, the trophoblast giant cells assume an invasive phenotype, thereby opening subluminal endometrial capillaries and establishing the first contact with maternal blood (Bevilacqua and Abrahamsohn, 1988, 1989). This process lasts for a few more days until the embryo is fully lodged in the uterine tissue. The placenta then starts the maturation process, which is characterized by the differentiation of the trophoblast cells to assume defensive, endocrine, immune regulatory, and nutritional properties (Adamson et al., 2002; Cross, 2005; Hu and Cross, 2009).

Evidence from a previous study in humans showed that vaccination results in viremia from the second to the sixth day after administration (Reinhardt et al., 1998). In this context, it is possible that in our experiments, maternal viremia had occurred at the phase of trophoblast immaturity soon after implantation. This might foster a condition of viral access to the embryonic tissues, boosting reactions not found when the vaccine is administered in later stages of pregnancy. In summary, on gd5.5 and the subsequent few days, the trophoblast giant cells may not be mature or differentiated enough to act as a barrier to viral passage. Based on this, vaccination in the later stages of gestation (gd11.5) might be related to full placental differentiation and ability for efficient activation of antiviral mechanisms. In contrast, the lack of contact between maternal blood and the embryo during the early stages of development, when the mothers were inoculated on gd0.5 may be the critical factor that prevented adverse fetal outcomes in this group. In both cases, our results over these periods are consistent with the findings of vaccination studies in humans.

The disparity with human vaccination data, however, occurred when vaccine inoculation was performed specifically during embryo implantation (day 5.5 of gestation), which resulted in early and late losses (stillbirths).

Studies assessing YF vaccination during early stages of pregnancy also reported a trend toward increased odds of several adverse events (miscarriages, premature births, and low birth weight) when women inadvertently received the vaccine through mass vaccination programs (Tsai, 1993, 2006; Nishioka et al., 1998). The deleterious effect on gestation outcome and fetal development, however, was considered within the expected population indices (Nasidi et al., 1993; Robert et al., 1999; Suzano et al., 2006; D’Acremont et al., 2008). As mentioned earlier, one possible explanation for this disparity may be the organization of the maternal-fetal barrier in its early stages, as having species-specific functional and structural aspects (Georgiades et al., 2002) may determine the passage of the virus to the fetal organism in rodents, but not in humans. In addition, the discrepancy may in part be due to the impossibility of determining in which gestational phase the vaccine has been administered. Peri-implantation losses in humans are hardly detectable, and hence, they are usually not reported and considered for statistical analysis reported in the literature.

The incidence of growth restriction and stillbirths in mothers vaccinated on gd5.5 may be based on several mechanisms, alone or in association. Viral access and lodgment in the developing mouse placenta may be a major factor. Although, morphologically, we did not see any placental damage or placental maturation defects, the immunoreactivity to the vaccine virus at the spongiotrophoblast and labyrinthine zones (also detected by PCR) may represent early access (gd5.5) and further colonization leading to different degrees of placental function impairment.

Virus immunolocalization graded from placental and fetal tissues with occasional antibody reactions (in the live fetuses) to areas of extensive antibody reactivity (in stillbirths), suggesting that the degree of placental/fetal infection might be related to fetal death.

Another possibility is the commitment of fetal metabolism when the placental barrier has not been able to prevent the passage of the virus. YF infection is characterized by a viral viscerotropism in which the liver and nervous system can be aggressively infected, as reported in humans who have succumbed to the infection post-vaccination (Monath, 2008; Martins et al., 2015; Monath and Vasconcelos, 2015). Our immunolocalization and PCR results also detected the presence of the vaccine virus and activity in the nervous tissues of stillborn fetuses, which may have contributed to the impairment of this system and fetal death as a direct cytopathic effect.

The fetal and maternal immune environment may also be an important factor in determining fetal infection when vaccination occurred on gd5.5. There is a well-orchestrated pro- and anti-inflammatory cytokine network locally produced to modulate the complex process of implantation (Dey et al., 2004; Chaouat et al., 2007). On the other hand, YF vaccination also induces a proinflammatory response, where TNF-α, among other cytokines seems to be a key factor (Monath and Vasconcelos, 2015). This may lead to an immune overreaction with the release of proinflammatory cytokines and other mediators of the innate immune system into the fetal circulation, resulting in outcomes such as early embryo deaths and low birth weight in the surviving fetuses (Chaouat et al., 2007; Chattopadhyay et al., 2010; Kurtis et al., 2011). Recent evidence has also shown that pregnant rat females with Zika virus infection exhibited a robust inflammatory response, including critical cytokines and chemokines, regardless of the mother’s response to the virus (Khaiboullina et al., 2019). A similar response is also observed in Zika virus infection in pregnant Rhesus monkeys (Hirsch et al., 2018). From this perspective, the plethora of immune factors that are triggered by viral infections may also have been the cause of the gestational outcomes obtained in this study, a question that deserves further research effort.

This study has no exact answers as to why this does not occur at the same frequency in humans. The greater fragility of the trophoblastic barrier in the early stages of gestation in mice may also be an essential factor. Protective neutralizing antibodies were found 14 days after vaccination in humans (Kohler et al., 2012), which were transferred to the embryo/fetus throughout the term of the human pregnancy, limiting viral growth and its deleterious effects. However, in this study, the short gestational period associated with the interval between vaccination and sacrifice of the mice (gd5.5 to gd18.5) might not be sufficient to transfer appropriate protective IgG to the fetal organism.

In summary, our results showed that mouse vaccination does not change gestational parameters when administered in early or mid-gestation. Adverse outcomes such as fetal growth restriction and increased rate of mortality could be observed only when vaccination occurred during the embryo implantation period. The localization of virus particles in the placenta and fetus indicates that YF vaccine virus may have crossed the placental barrier in a stochastic process. In living fetuses, the presence of the virus was limited or absent, whereas in stillbirths, the immunoreactivity and the viral load were high in the placenta and fetal organs. The heterogeneity of responses suggests that the stage of embryo implantation represents a window of susceptibility in which vaccination and associated immune response may interfere with the course of gestation.

## Conclusion

The yellow fever vaccine virus passed the placental barrier only when administered during embryo implantation, inducing fetal growth restriction and increased fetal mortality rate.

## Data Availability Statement

The datasets generated for this study are available on request to the corresponding author.

## Ethics Statement

The animal study was reviewed and approved by the Ethics Committee for Animal Research (CEEA) of Biomedical Sciences Institute of the University of São Paulo, Brazil (no. 126/37 book2) – Institute of Biomedical Sciences – USP.

## Author Contributions

FS and EB wrote the draft of the manuscript. EB and HS designed the study. All authors except HS participated in data collection. Data analysis was conducted by FS and FM, who vouch for the findings.

## Conflict of Interest

The authors declare that the research was conducted in the absence of any commercial or financial relationships that could be construed as a potential conflict of interest.

## Footnotes

1 http://www.ncbi.nlm.nih.gov/BLAST/
